# Association of Cerebrospinal Fluid Parameters and Neurofilament Light Chain With Retinal Nerve Fiber Layer Thickness in Multiple Sclerosis

**DOI:** 10.3389/fneur.2022.814734

**Published:** 2022-03-07

**Authors:** Nik Krajnc, Patrick Altmann, Katharina Riedl, Christoph Mitsch, Thomas Berger, Fritz Leutmezer, Paulus Rommer, Berthold Pemp, Gabriel Bsteh

**Affiliations:** ^1^Department of Neurology, Medical University of Vienna, Vienna, Austria; ^2^Department of Ophthalmology, Medical University of Vienna, Vienna, Austria

**Keywords:** multiple sclerosis, biomarker, optical coherence tomography, RNFL, cerebrospinal fluid, neurofilament

## Abstract

**Introduction:**

Multiple sclerosis (MS) pathophysiology comprises both inflammatory and neurodegenerative characteristics. Cerebrospinal fluid (CSF) analysis allows for assessment of inflammation while neurofilament light chain can indicate neuroaxonal damage. Retinal thinning is a robust prognostic biomarker for neurodegeneration in MS. To date, an association between CSF parameters upon MS diagnosis and retinal thinning has not been investigated.

**Aims and Objectives:**

We aimed to determine whether CSF parameters are associated with the evolution of retinal layer thinning in people with MS (pwMS).

**Methods:**

For this longitudinal observational study, we investigated pwMS from the Vienna MS database (VMSD), who had undergone (1) a diagnostic lumbar puncture (LP) between 2015 and 2020, and (2) simultaneous optical coherence tomography (OCT) and/or (3) a follow-up OCT scan. Linear stepwise regression models were calculated with OCT parameters (peripapillary retinal nerve fiber layer [pRNFL] thickness at LP and at follow-up, annualized loss of pRNFL thickness [aLpRNFL]) as a dependent variable, and CSF parameters (white blood cell [WBC] count, total protein [_CSF_TP], CSF/serum albumin ratio [Q_alb_], intrathecal synthesis of immunoglobulins, neurofilament light chain [NfL] in both CSF and serum [_CSF_NfL/sNfL]) as independent variables adjusted for age, sex, and disease duration.

**Results:**

We analyzed 61 pwMS (median age 30.0 years [interquartile range 25.5–35.0], 57.4% female, median disease duration 1.0 month [IQR 0–2.0] before LP, median follow-up 1.9 years [IQR 1.1–3.5]). _CSF_NfL and sNfL measurements were available in 26 and 31 pwMS, respectively. pRNFL thickness at LP was inversely associated with the CSF WBC count (β = −0.36; 95% CI −0.51, −0.08; *p* = 0.008). We did not find any association between other CSF parameters, including _CSF_NfL, sNfL, and aLpRNFL.

**Conclusions:**

Increased WBC count as an indicator of acute inflammation and blood-brain-barrier breakdown seems to be associated with the amount of retinal thickness already lost at the time of LP. However, neither routine CSF parameters nor a singular NfL measurement allows the prediction of future retinal thinning.

## Introduction

Multiple sclerosis (MS) is an inflammatory demyelinating disease of the central nervous system (CNS) that represents the most common neurological disease in young adults (1). The current prevailing concept of MS pathophysiology suggests a disease process of both inflammatory and neurodegenerative characteristics. While neurodegenerative processes are predominating in later phases, it seems that neurodegeneration starts early on and, thus, influences long-term prognosis. Hence, there is an urgent need for surrogate markers that allow for the reliable evaluation of MS-associated neurodegeneration throughout the disease course as well as at the time of diagnosis (2). Current concepts for measuring neuroinflammation and neurodegeneration upon MS diagnosis include optical coherence tomography (OCT), standard cerebrospinal fluid (CSF) parameters, and neurofilament light chain (NfL). OCT is a non-invasive and accessible technique that uses near-infrared light to create high-resolution cross-sectional images of retinal layers (3). The thickness of the peripapillary retinal nerve fiber layer (pRNFL) and the macular ganglion cell-inner plexiform layer (GCIPL) are both robust indicators of neuroaxonal degeneration in MS (4). Annualized loss of both pRNFL (aLpRNFL) and GCIPL (aLGCIPL) thickness exceeding 1.5 and 1.0 μm/year, respectively, predict disability progression at considerable sensitivity and specificity (5–7). CSF analysis is a standard diagnostic procedure in the initial evaluation of MS in Europe. The leukocyte count (white blood cell [WBC] count, cell count) in the CSF as a non-specific indicator of acute inflammation is increased up to 50 cells/μl in about half of people with MS (pwMS) (8). The hallmark of typical CSF changes in MS is the increased production of intrathecal immunoglobulins (Ig), indicated by an increased IgG index or the presence of CSF oligoclonal bands (OCB) (9–11). Over the past few years, neurofilament light chain (NfL) in the blood and CSF have emerged as a promising biomarker in MS (12). As a major component of the neuronal cytoskeleton, NfL is released into the CSF and blood upon neuroaxonal injury (13). NfL concentrations are associated with the occurrence of relapses and neurological disability as well as MS lesions and brain atrophy on MRI (13, 14).

To date, no studies have been published concerning the prognostic impact of the described CSF parameters on retinal layer thinning. With this study, we investigated whether conventional CSF parameters and NfL are associated with pRNFL at baseline and follow-up in a cohort of newly diagnosed pwMS.

## Methods

### Patients and Definitions

For this longitudinal observational study, we extracted serum and CSF from patients in the Vienna MS database (VMSD) diagnosed with relapsing-remitting MS (RRMS) according to the 2017 McDonald criteria, aged between 18 and 65 years, who had a diagnostic lumbar puncture (LP) between January 1, 2015 and December 31, 2019, and (1) OCT at LP, and/or (2) follow-up OCT.

We documented age and disease duration at baseline as well as the duration of the follow-up period, defined by an interval between the diagnostic LP and the last OCT scan. A relapse was defined as patient-reported symptoms and objectively confirmed neurological signs typical of an acute CNS inflammatory demyelinating event with a duration of at least 24 h in the absence of fever or infection and separated from the last relapse by at least 30 days (15). The Expanded Disability Status Scale (EDSS) score was obtained at baseline and follow-up (16), with EDSS progression defined as a confirmed EDSS increase of ≥1.5 points in patients with a baseline EDSS of 0, an increase of ≥1.0 point in patients with a baseline EDSS of 1.0–5.5, or an increase of ≥0.5 point in patients with a baseline EDSS of ≥6.0.

The patients' disease-modifying treatment (DMT) status was classified as follows: (1) “no DMT” defined as patients receiving no DMT between diagnostic LP and the last OCT scan; (2) “moderately effective DMT (M-DMT)” defined as patients receiving either interferon-beta preparations, glatiramer acetate, dimethyl fumarate or teriflunomide; or (3) “highly effective DMT (H-DMT)” defined as patients receiving either natalizumab, fingolimod, alemtuzumab, cladribine, ocrelizumab, or rituximab. We also defined “escalation (ESC-DMT)” as patients switching from no DMT to moderately effective DMT or from moderately effective DMT to highly effective DMT between the diagnostic LP and the last OCT scan.

### CSF Parameters

In all patients, routine LP was performed at baseline as part of the routine diagnostic work-up following written informed consent. The routine CSF parameters included: WBC count, total protein (_CSF_TP), CSF/serum albumin ratio (*Q*_alb_), intrathecal synthesis of IgA, IgM, and IgG including oligoclonal-IgG-bands (OCB).

### Optical Coherence Tomography

Optical coherence tomography (OCT) imaging was performed by experienced neuro-ophthalmologists at the Department of Ophthalmology and Optometry of the same institution using the same spectral-domain OCT (Spectralis OCT, Heidelberg Engineering, Heidelberg, Germany; software Heidelberg eye explorer software version 5.4.8.0) without pupil dilatation in a dark room on both eyes of each patient. For pRNFL measurement, a custom 3.4 mm ring scan (12°) centered on the optic nerve head was used (1,536 A-scans, automatic real-time tracking [ART]: 100 averaged frames) (17), Image processing was conducted semiautomated with manual correction of obvious errors. All examinations were checked for sufficient quality using the OSCAR-IB criteria (18). For patients without a history of optic neuritis (ON), pRNFL thickness was calculated as the mean of the values for both eyes. For patients with a history of unilateral ON, only the values of eyes without ON were used in the analyses.

Eyes suffering ON during the observation period were excluded from the longitudinal part of the study and only the values of eyes without ON during the observation period were used for the calculation of retinal thinning in the analyses (6). To identify subclinical ON at baseline, we used interocular asymmetry with cut-off values of ≥5 μm for pRNFL (19, 20). To identify subclinical ON, during the course of the study, we used interocular asymmetry in retinal thinning (i.e., an increase in the inter-eye difference in pRNFL compared to the prior OCT) with a cut-off value of ≥5 μm. In these cases, we used only the eye with the higher value. aLpRNFL was calculated by individual linear regression models as the slope of the regression line best fitter to all pRNFL measurements over the observation period (6, 21). Based on the previous studies, we dichotomized patients into two groups using a pRNFL cut-off value of 88 μm and an aLpRNFL cut-off value of 1.5 μm/year (6, 21). Patients with diagnoses of ophthalmological, neurological, or drug-related causes of vision loss or retinal damage not attributable to MS were excluded (22). The investigators performing the OCT were blinded to clinical parameters and vice versa. The quantitative OCT study results were reported using the revised Advised Protocol for OCT Study Terminology and Elements (APOSTEL 2.0) recommendations (23).

### Neurofilament Light Chains

Serum and CSF samples were extracted from our neurological biobank where aliquots are stored at −70°C in accordance with international consensus guidelines (24, 25). Concentrations of sNfL and _CSF_NFL were measured on the SR-X analyzer (Quanterix International, Billerica, MA, USA) using commercially available Simoa NF-light kits and following the manufacturer's instructions. The investigators performing the _CSF_NfL and sNfL testing were blinded to the clinical and OCT parameters, and vice versa. The samples were analyzed as duplicates. Only samples yielding a coefficient of variance (CV) of <0.2 were included in this study. Sensitivity analyses for missing NfL values were performed as a separate analysis of parameters for subjects with available and subjects with missing NfL values.

### Ethics

The study was approved by the ethics committee of the Medical University of Vienna, Austria (EK1446/2021).

### Statistics

Statistical analysis was performed using SPSS 26.0 (SPSS Inc, Chicago, IL, USA). Categorical variables were expressed in frequencies and percentages, continuous variables as mean and SD or median and interquartile range (IQR) as appropriate. Continuous variables were tested for normal distribution by the Kolmogorov–Smirnov test with Lilliefors correction. Univariate comparisons were done by a chi-square test, Mann-Whitney U-test, or independent *t-*test as appropriate.

First, we performed a univariate analysis with Pearson and Spearman correlation analyses on the CSF parameters (WBC, _CSF_TP, *Q*_alb_, intrathecal synthesis of IgA, IgM and IgG, OCB) and OCT parameters (pRNFL at LP and at last follow-up, aLpRNFL). Linear stepwise regression models were fitted with the OCT parameters (pRNFL, aLpRNFL) as the dependent variable and the CSF parameters as independent variables adjusted for age, sex, and disease duration. Second, the _CSF_NfL, sNfL, and OCT parameters were univariately analyzed by Pearson or Spearman correlation analyses. Linear stepwise regression models were fitted with the OCT parameters as the dependent variable, and the CSF parameters, _CSF_NfL, and sNfL as independent variables (in separate models to avoid collinearity) adjusted for age, sex, and disease duration. As covariates, the number of relapses and disability progression during follow-up as well as the DMT group was included. A value of *p* < 0.05 was considered statistically significant. All multiple analyses were corrected using the Bonferroni method.

## Results

### Demographics

**Figure 1:**
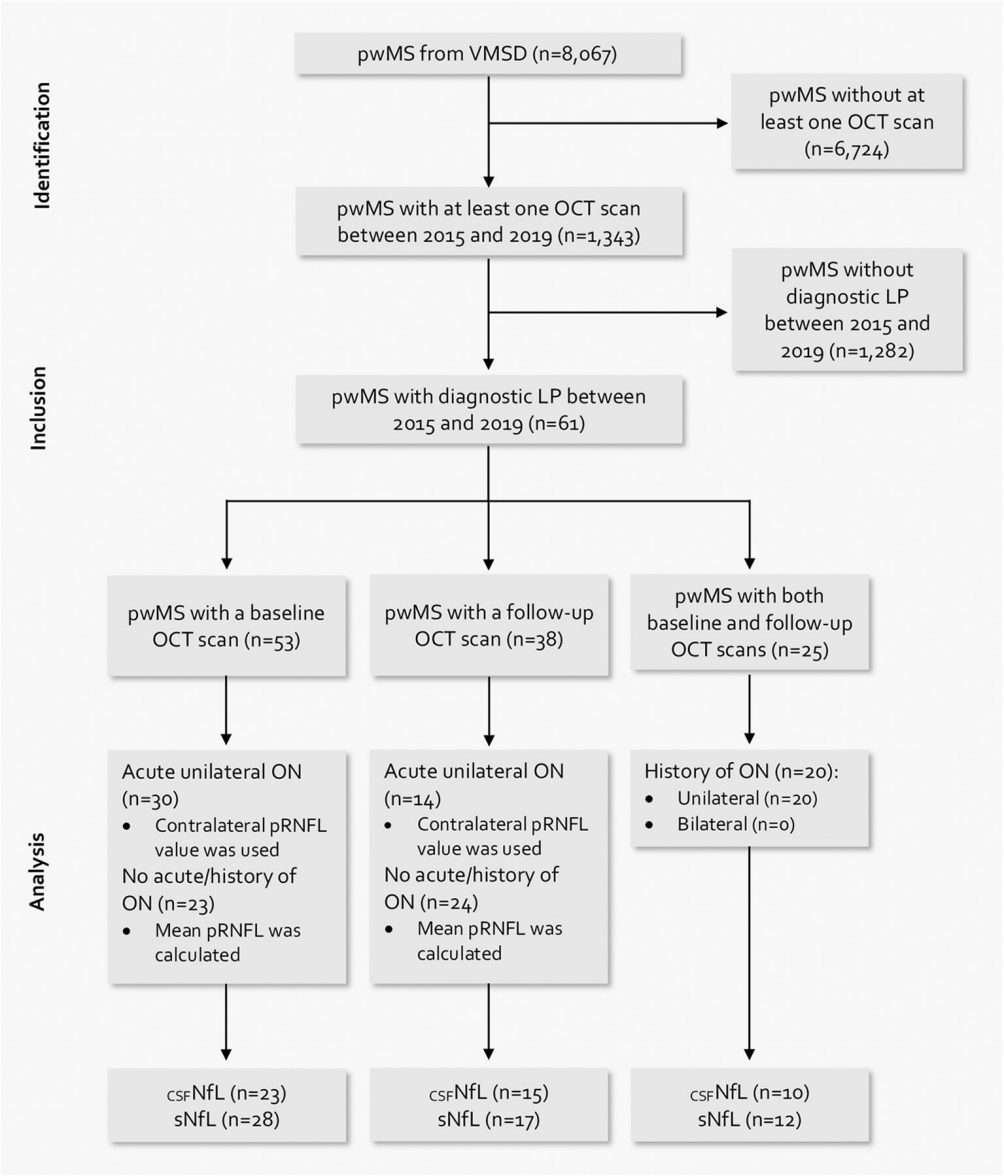
Flow chart of patient selection based on the inclusion and exclusion criteria.

We included 61 pwMS in the analysis (Figure 1). In total, 53 (86.9%) had a baseline OCT scan, 38 (62.3%) had a follow-up OCT scan, and 25 (41.0%) had both a baseline and a follow-up OCT scan. Baseline CSF samples for _CSF_NfL measurements were available in 26 (42.6%) patients, and baseline serum samples for sNfL measurements were available in 31 (50.8%) patients. Sensitivity analyses regarding missing NfL values did not show a significant effect of missing values on the reported results. The demographics and clinical characteristics of the study cohort are given in Table 1. Oligoclonal bands (OCB) were positive in 58 patients, 2 patients were negative for OCB, and the OCB results for one patient were missing. At baseline, no patient received DMT.

**Table 1:** Demographics and clinical characteristics of the study cohort.

	**Study cohort (*n* = 61)**	**Baseline cohort (*n* = 53)**	**Follow-up cohort (*n* = 38)**	**aLpRNFL cohort (*n* = 25)**
Female^a^	35 (57.4)	29 (54.7)	24 (63.2)	13 (52.0)
Age (years)^b^	30.0 (25.5–35.0)	29.0 (24.3–32.5)	29.5 (26.0–37.0)	27.0 (25.0–34.5)
Disease duration (months)^b^	1 (0–2)	1 (0–2)	NA	NA
Follow-up period (years)^b^	NA	NA	1.9 (0.9–3.3)	1.9 (1.1–3.5)
EDSS at baseline^b^	NA	1.0 (0–2.0)	NA	0.5 (0–1.9)
EDSS at last follow-up^b^	NA	NA	1.0 (0–2.0)	1.0 (0–2.0)
Relapse-free patients during follow-up^a^	NA	NA	1 (2.6)	0 (0.0)
ARR^b^	NA	NA	0.8 (0.6–1.2)	0.8 (0.5–1.4)
EDSS progression during follow-up^a^	NA	NA	9 (23.7)	7 (28.0)
**DMT**				
No DMT^a^	31 (50.8)	53 (100.0)	9 (23.7)	7 (28.0)
M-DMT^a^	21 (34.4)	0 (0)	20 (52.6)	11 (44.0)
Interferon beta	2 (3.2)	NA	NA	NA
Glatiramer acetate	10 (16.4)	NA	NA	NA
Dimethyl fumarate	9 (14.8)	NA	NA	NA
H-DMT^a^	9 (14.8)	0 (0)	8 (21.1)	7 (28.0)
Fingolimod	2 (3.3)	NA	NA	NA
Natalizumab	2 (3.3)	NA	NA	NA
Alemtuzumab	1 (1.6)	NA	NA	NA
Rituximab	3 (4.9)	NA	NA	NA
Ofatumumab	1 (1.6)	NA	NA	NA
ESC-DMT^a^	1 (1.6)	0 (0)	1 (2.6)	0 (0.0)
**OCT parameter**				
pRNFL at baseline (μm)^c^	NA	97.0 (10.9)	NA	97.2 (8.9)
pRNFL at last follow up (μm)^c^	NA	NA	95.6 (10.0)	96.1 (6.5)
aLpRNFL (μm/year)^c^	NA	NA	NA	−0.6 (1.5)

*aLpRNFL, annualized loss of peripapillary retinal nerve fiber layer; ARR, annualized relapse rate; DMT, disease modifying treatment; EDSS, Expanded Disability Status Scale; NA, not applicable; OCT, optical coherence tomography; pRNFL, peripapillary retinal nerve fiber layer*.

a *Number (percentage)*,

b *Median and interquartile range*,

c *Mean and standard deviation*.

### pRNFL at Baseline

**Figure 2:**
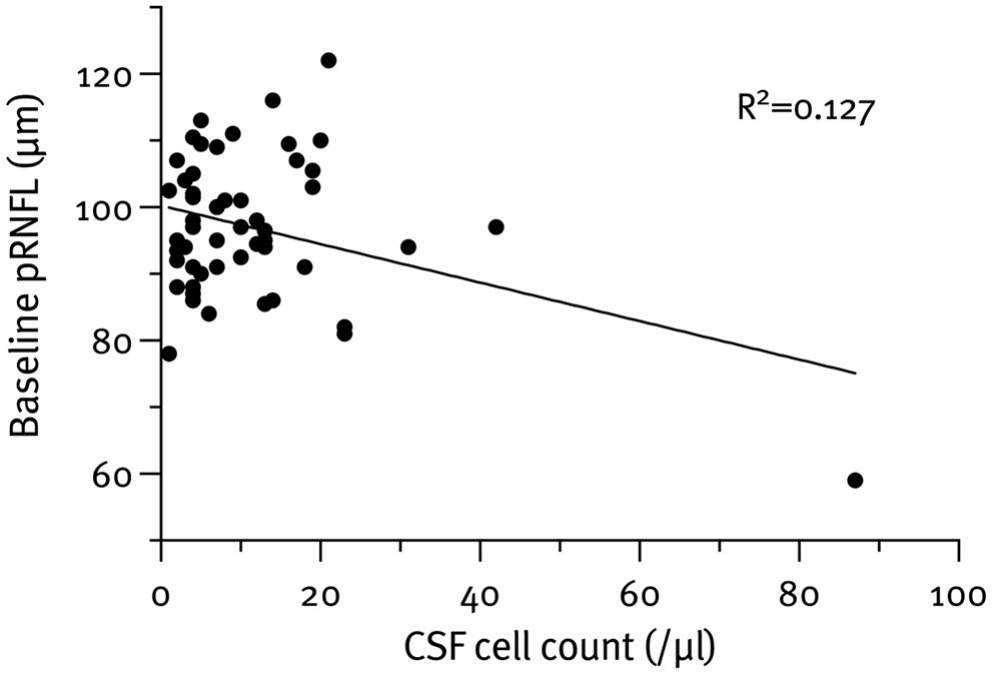
Cerebrospinal fluid (CSF) cell count (WBC) correlated with the peripapillary retinal nerve fiber layer (pRNFL) thickness at baseline (*p* = 0.008).

The mean pRNFL thickness at baseline was 97.0 μm ([SD] = 10.9). In total, 11 (20.8%) patients had a pRNFL thickness ≤ 88 μm at baseline. We detected no differences in the CSF parameters between these two groups. In the multivariable analyses, pRNFL thickness at baseline was associated with the WBC count (β = −0.36; 95% CI −0.51, −0.08; *p* = 0.008) but not with any of the other CSF parameters (Figure 2).

### pRNFL at Follow-Up

The mean pRNFL thickness at follow-up was 95.6μm (SD = 10.0). In total, 7 (18.4%) patients had a pRNFL thickness ≤ 88 μm at follow-up. We detected no differences in CSF parameters between these two groups, and there was no association between pRNFL thickness and CSF parameters.

### aLpRNFL

The median follow-up period was 1.9 (IQR 1.1–3.5) years. The mean aLpRNFL was −0.6 (SD 1.5) μm/year with 6 (24.0%) patients showing an aLpRNFL ≥1.5 μm/year. In our sample, the aLpRNFL correlated with both the IgA ratio (*r*_*s*_ = 0.629, *p* = 0.016) and IgA index (*r*_*s*_ = 0.678, *p* = 0.008) but not with other CSF parameters (Table 2). Multivariate regression models revealed no association between CSF parameters and aLpRNFL.

**Table 2:** Univariate correlation between cerebrospinal fluid (CSF) parameters and annualized loss of peripapillary retinal nerve fiber layer (aLpRNFL).

	**Study cohort (*n* = 61)**	**aLpRNFL ≥1.5μm/y (*n* = 6)**	**aLpRNFL <1.5μm/y (*n* = 19)**	**Correlation with aLpRNFL**
WBC count (/μl)^a^	7 (4–13.5)	8 (6.5–12.5)	7 (4–19)	*r_*s*_* = −0.025
Total protein level (mg/dl)^b^	35.6 (12.4)	39.9 (11.1)	35.0 (17.3)	*r* = −0.017
Serum IgG level (mg/dl)^b^	944.95 (180.42)	855.33 (223.40)	955.42 (162.73)	*r* = −0.240
CSF IgG level (mg/dl)^a^	3.33 (2.19–4.52)	3.19 (2.13–4.16)	3.53 (1.56–6.39)	*r_*s*_* = −0.213
IgG ratio (/1,000)^a^	3.57 (2.65–4.92)	3.46 (3.16–4.10)	3.57 (1.78–6.46)	*r_*s*_* = −0.178
IgG index^a^	0.70 (0.61–1.12)	0.64 (0.55–0.94)	0.76 (0.66–1.23)	*r_*s*_* = −0.282
Serum IgA level (mg/dl)^a^	153 (133–239.5)	148.5 (106.3–379.3)	204 (115–271)	*r_*s*_* = −0.026
CSF IgA level (mg/dl)^a^	0.48 (0.27–0.62)	0.25 (0.14–0.90)	0.46 (0.12–0.98)	*r_*s*_* = −0.407
IgA ratio (/1,000)^a^	1.90 (1.26–2.81)	1.30 (0.90–2.10)	2.63 (0.84–4.55)	*r_*s*_* = −0.629^**^
IgA index^a^	0.32 (0.24–0.44)	0.23 (0.22–0.30)	0.30 (0.26–0.70)	*r_*s*_* = −0.678^***^
Serum IgM level (mg/dl)^a^	105 (69.5–147)	63.5 (45.8–85.3)	114 (76–169)	*r_*s*_* = −0.176
CSF IgM level (mg/dl)^a^	0.06 (0.03–0.08)	0.05^*^	0.03^*^	*r_*s*_* = 0.316
IgM ratio (/1,000)^a^	0.44 (0.27–0.87)	0.54^*^	0.12^*^	*r_*s*_* = 0.700
IgM index^a^	0.08 (0.06–0.18)	0.12^*^	0.05^*^	*r_*s*_* = 0.500
Albumin ratio (/1,000)^a^	4.85 (3.12–6.18)	5.14 (3.66–7.58)	3.26 (2.65–5.69)	*r_*s*_* = 0.102
_CSF_NfL (pg/ml)^a^	798.8 (426.3–1,507.6)	830.7 (300.5–1,387.1)	497.3 (447.9–752.7)	*r_*s*_* = 0.146
sNfL (pg/ml)^a^	10.6 (7.1–20.2)	6.9 (4.8–45.8)	8.0 (4.3–12.6)	*r_*s*_* = 0.159

*aLpRNFL, annualized loss of peripapillary retinal nerve fiber layer; CSF, cerebrospinal fluid; NfL, neurofilament light chain; WBC, white blood cell*.

a *Median and interquartile range*,

b *Mean and standard deviation*.

* *Sample size too small to calculate distributions*,

** *p < 0.05*,

*** *p < 0.01*.

### NfL Concentrations

**Figure 3:**
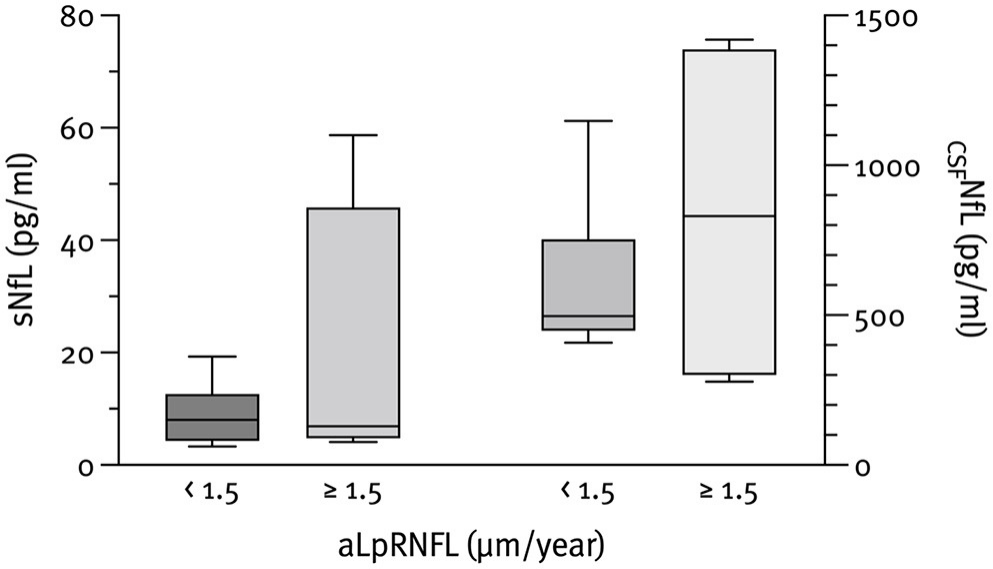
Baseline CSF neurofilament light chain (_CSF_NfL) (*n* = 10) and serum neurofilament light chain (sNfL) (*n* = 12) concentrations according to the rate of annualized loss of pRNFL (aLpRNFL).

**Figure 4:**
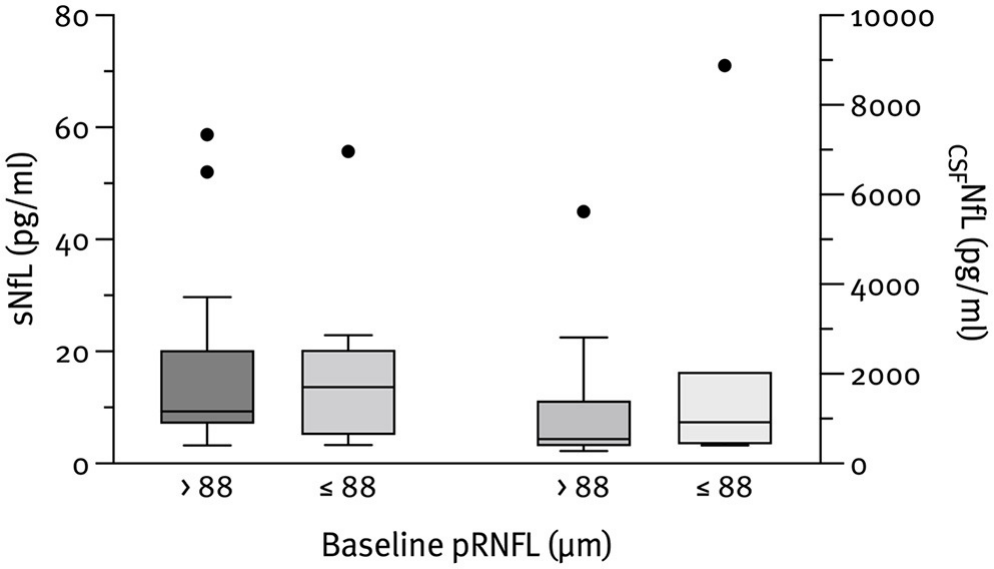
Baseline _CSF_NfL (*n* = 23) and sNfL (*n* = 28) concentrations according to the pRNFL thickness at baseline. Outliers present the data outside 1.5 times the interquartile range above the upper and below the lower quartile.

The median NfL concentrations at baseline were 798.8 pg/ml (IQR 426.3–1,507.6) for CSF and 10.6 pg/ml (IQR 7.1–20.2) for serum. As expected, _CSF_NfL correlated significantly with sNfL (*r*_*s*_ = 0.785; *p* < 0.001); however, no correlation with pRNFL or aLpRNFL was found. Patients with evidence of neuroaxonal loss (characterized by an aLpRNFL ≥1.5 μm/year) had higher concentrations of _CSF_NfL (830.7 vs. 497.3 pg/ml) and sNfL (6.9 vs. 8.0 pg/ml) compared to patients with aLpRNFL <1.5 μm/year, but these differences were not statistically significant (*p* = 0.999 and *p* = 0.933, respectively) (Figure 3). We also analyzed NfL concentrations for patients below and above the cohort's median pRNFL thickness of 88 μm but found no significant difference between these groups (Figure 4).

## Discussion

In this longitudinal observational study of 61 pwMS, we aimed to determine an association between conventional and novel CSF parameters at the time of diagnosis and OCT parameters at baseline and follow-up (median 1.9 years). This is important, as retinal layer thinning on OCT is a relevant biomarker for MS-associated neurodegenerative changes, such as neuroaxonal loss, physical and cognitive disability, and brain atrophy (22, 26–28).

Out of all classical CSF parameters investigated, WBC count emerged as the sole statistically significant predictor of pRNFL thickness at baseline. That being said, the effect size was moderate at best and only explained about 13% of the pRNFL thickness variation.

Importantly, the CSF cell count was not associated with pRNFL thickness at follow-up or thinning of pRNFL during follow-up. The observation that WBC count is associated with pRNFL thickness at the time of LP but not with further pRNFL thinning suggests that WBC count is reflective of short-term inflammatory activity rather than long-term disease progression, i.e., neuroaxonal damage. This is aligned with evidence indicating that increased WBC is associated with faster conversion from clinically isolated syndrome (CIS) to clinically definite MS but only very weakly with EDSS worsening (29, 30).

This was the first published study to investigate the associations between NfL in CSF and retinal layer thinning. We did not find any significant association, even though we observed a trend toward increased CSF NfL levels and both lower pRNFL thickness at baseline, as well as increased aLpRNFL. Previous studies have shown an association between NfL levels in serum and both pRNFL thickness and aLpRNFL (31). As serum and CSF NfL levels are tightly correlated, the lack of a significant association may be explained by various factors. Our cohort consisted mostly of newly diagnosed patients, thus, the degree of neuroaxonal damage was inherently low, narrowing the potential margin of prediction. Also, the majority of patients (29/38; 76.3%) were placed on DMT early on, which may negate the predictive capacity of NfL. Furthermore, it is important to bear in mind that NfL levels are somewhat volatile and one-time sampling combined with a low prognostic margin may lead to statistically unfavorable results (31).

Our results are limited with respect to the small sample size. Data on aLpRNFL was only available for 25 patients with only 6 of them showing signs of progression. We did not include GCIPL thickness which is reported to be even a more sensitive biomarker of disease progression in MS (5, 32, 33). Also, paired serum and CSF samples for NfL analysis were not available for all subjects. We tried to overcome this predicament by performing sensitivity analyses for the missing NfL values and found no significant unfavorable effect. Besides, due to the lack of sufficient MRI data, the optic chiasm involvement was not excluded. However, the potential impact of optic chiasm involvement is probably low as this is—contrary to neuromyelitis optica spectrum disorder—rare in MS.

In conclusion, we showed some evidence that from all CSF parameters obtained at diagnosis, the WBC count remained the only parameter to determine decreased retinal thickness. In our model, NfL concentrations in both serum and CSF upon diagnosis were not robust enough to predict subsequent retinal thinning.

## Data Availability Statement

The original contributions presented in the study are included in the article/supplementary material, further inquiries can be directed to the corresponding author/s.

## Ethics Statement

The studies involving human participants were reviewed and approved by the Ethics Committee of the Medical University of Vienna, Austria (EK1446/2021). Written informed consent for participation was not required for this study in accordance with the national legislation and the institutional requirements.

## Author Contributions

NK: acquisition of data, data management, statistical analysis and interpretation of data, and drafting of the manuscript. PA: acquisition of data, data management, interpretation of data, and drafting of the manuscript. KR, CM, PR, FL, TB, and BP: acquisition of data and critical revision of the manuscript for intellectual content. GB: study concept and design, acquisition of data, interpretation of data, study supervision, and critical revision of the manuscript for intellectual content. All authors contributed to the article and approved the submitted version.

## Funding

This research was funded by the Medical University of Vienna.

## Conflict of Interest

NK has participated in meetings sponsored by, received speaker honoraria or travel funding from Roche, Novartis, and Merck, and holds a grant for a Multiple Sclerosis Clinical Training Fellowship Programme from the European Committee for Treatment and Research in Multiple Sclerosis (ECTRIMS). PA has participated in meetings sponsored by, received speaker honoraria or travel funding from Biogen, Merck, Roche, Sanofi-Genzyme, and Teva, and received honoraria for consulting from Biogen. He received a research grant from Quanterix International and was awarded a combined sponsorship from Biogen, Merck, Sanofi-Genzyme, Roche, and Teva for a clinical study. CM has received honoraria for consultancy/speaking (incl. funds for e-learning modules) from Bayer, Novartis, and Takeda. PR has received honoraria for consultancy/speaking from AbbVie, Allmiral, Alexion, Biogen, Merck, Novartis, Roche, Sandoz, Sanofi Genzyme, has received research grants from Amicus, Biogen, Merck, Roche. FL received honoraria (lectures, advisory boards, consultations) from pharmaceutical companies marketing treatments for MS: Alexion, Almirall, Bayer, Biogen, Celgene, MedDay, Merck, Novartis, Octapharma, Roche, Sanofi-Genzyme, Teva. TB has participated in meetings sponsored by and received honoraria (lectures, advisory boards, consultations) from pharmaceutical companies marketing treatments for MS: Almirall, Biogen, Bionorica, Celgene/BMS, GSK, Janssen-Cilag, MedDay, Merck, Novartis, Roche, Sandoz, Sanofi-Genzyme, Teva. His institution has received financial support in the past 12 months by unrestricted research grants Biogen, Celgene/BMS, Merck, Novartis, Sanofi Aventis, Teva and for participation in clinical trials in multiple sclerosis sponsored by Alexion, Biogen, Merck, Novartis, Octapharma, Roche, Sanofi-Genzyme, Teva. BP has received honoraria for consulting from Novartis. GB has participated in meetings sponsored by, received speaker honoraria or travel funding from Biogen, Celgene, Lilly, Merck, Novartis, Sanofi-Genzyme, and Teva, and received honoraria for consulting Biogen, Celgene, Novartis, Sanofi-Genzyme, Roche, and Teva. The remaining author declares that the research was conducted in the absence of any commercial or financial relationships that could be construed as a potential conflict of interest.

## Publisher's Note

All claims expressed in this article are solely those of the authors and do not necessarily represent those of their affiliated organizations, or those of the publisher, the editors and the reviewers. Any product that may be evaluated in this article, or claim that may be made by its manufacturer, is not guaranteed or endorsed by the publisher.

## Abbreviations

aLGCIPL: annualized loss of ganglion cell-inner plexiform layer
aLpRNFL: annualized loss of peripapillary retinal nerve fiber layer
ARR: annualized relapse rate
CIS: clinically isolated syndrome
CSF: cerebrospinal fluid
CV: coefficient of variance
DMT: disease modifying treatment
EDSS: Expanded Disability Status Scale
ESC-DMT: escalation of disease-modifying treatment
GCIPL: ganglion cell-inner plexiform layer
H-DMT: highly effective disease-modifying treatment
IQR: interquartile range
LP: lumbar puncture
M-DMT: moderately effective disease-modifying treatment
MS: multiple sclerosis
NfL: neurofilament light chain
OCB: oligoclonal bands
OCT: optical coherence tomography
ON: optic neuritis
pRNFL: peripapillary retinal nerve fiber layer
pwMS: patients with multiple sclerosis
Q_alb_: CSF/serum albumin ratio
SD: standard deviation
TP: total protein
VMSD: Vienna Multiple Sclerosis Database
WBC: white blood cell.
